# Preventing Occupational Tuberculosis in Health Workers: An Analysis of State Responsibilities and Worker Rights in Mozambique

**DOI:** 10.3390/ijerph17207546

**Published:** 2020-10-16

**Authors:** Regiane Garcia, Jerry M. Spiegel, Annalee Yassi, Rodney Ehrlich, Paulo Romão, Elizabete A. Nunes, Muzimkhulu Zungu, Simphiwe Mabhele

**Affiliations:** 1 School of Population and Public Health, University of British Columbia, Vancouver, BC V6T 1Z3, Canada; annalee.yassi@ubc.ca; 2 Division of Occupational Medicine, School of Public Health and Family Medicine, University of Cape Town, Rondebosch 7701, South Africa; rodney.ehrlich@uct.ac.za; 3 International Labour Organization, 688 Av. do Zimbábwe, Maputo, Mozambique; promaomoz@yahoo.com.br; 4 Department of Internal Medicine, Maputo Central Hospital, 364 Av. Agostinho Neto, Maputo 1100, Mozambique; dra.elizabete.nunes@gmail.com; 5 National Institute for Occupational Health, 25 Hospital St, Constitution Hill, Johannesburg 2000, South Africa; MuzimkhuluZ@nioh.ac.za; 6 School of Health Systems and Public Health, University of Pretoria, Pretoria 0002, South Africa; 7 International Labour Organization, Block C, Crestway Office Park, 20 Hotel St. Persequor, Pretoria 0020, South Africa; mabhele@ilo.org

**Keywords:** occupational health, health workers, rights, laws, governance, implementation science

## Abstract

Given the very high incidence of tuberculosis (TB) among health workers in Mozambique, a low-income country in Southern Africa, implementation of measures to protect health workers from occupational TB remains a major challenge. This study explores how Mozambique’s legal framework and health system governance facilitate—or hinder—implementation of protective measures in its public (state-provided) healthcare sector. Using a mixed-methods approach, we examined international, constitutional, regulatory, and policy frameworks. We also recorded and analysed the content of a workshop and policy discussion group on the topic to elicit the perspectives of health workers and of officials responsible for implementing workplace TB policies. We found that despite a well-developed legal framework and national infection prevention and control policy, a number of implementation barrier persisted: lack of legal codification of TB as an occupational disease; absence of regulations assigning specific responsibilities to employers; failure to deal with privacy and stigma fears among health workers; and limited awareness among health workers of their legal rights, including that of collective action. While all these elements require attention to protect health workers from occupational TB, a stronger emphasis on their human and labour rights is needed alongside their perceived responsibilities as caregivers.

## 1. Introduction

Throughout the 21st Century, tuberculosis (TB) has continued to be the leading cause of death due to infectious disease globally, killing 1.7 million people in 2018 [1]. The global rise of multidrug-resistant TB (MDR-TB) further threatens TB control [1,2]. Health workers involved in caring for patients with TB and MDR-TB have at least a two- to three-fold higher risk of contracting the disease than that of the general population [3,4,5,6,7]. This is especially problematic in low-income, high burden TB and TB-HIV co-infection settings such as the Republic of Mozambique in Southern Africa (our case study), where the annual incidence of TB is 551 per 100000, with an estimated 58% of these HIV-co-infected [1]. Mozambique lacks nationally representative data on TB rates among health workers [8,9,10]. However, a recent study conducted at Maputo Central Hospital, a public teaching quaternary care hospital, identified an annual incidence rate among health workers of 1676 per 100,000, consistent with the median incidence rate of 1180 per 100,000 reported in a meta-analysis of active TB among health workers in high-TB incidence countries [11]. Effective implementation of a workplace TB policy for health workers is thus urgently needed in Mozambique [11,12,13].

Interventions to protect health workers against occupational TB infection in Mozambique, as elsewhere, occur predominantly within the framework of infection prevention and control (IPC) [14]. However, despite the accumulation of scientific knowledge and the publication of international guidelines [15,16,17] implementation of IPC measures remains a major challenge in low-income, high TB burden settings [18,19].

In this article, we argue that an enabling legal and governance environment is needed if persistent occupational TB transmission in healthcare facilities is to be reduced [20]. Laws define rights and obligations with respect to implementation and accountability [21]. Governance is more broadly defined as the processes that “determine(s) who has power, who makes decisions, how other players make their voice heard, and how account is rendered” [22]. 

To date, legal studies of high TB burden settings have largely employed rules-based indicators, i.e., a focus on the existence of adequate rules and procedures [23] (pp. 86,87), e.g., to determine the extent to which countries have incorporated legal aspects of IPC international treaties into legislation, such as TB case-reporting and surveillance [24,25]. However, as research is lacking on the processes of implementing laws and the impact of such laws on health workers’ occupational health and safety, key questions remain unanswered. 

Using Mozambique’s public healthcare sector as a case study, our exploratory study employed an outcomes-based approach, i.e., a focus on the implementation or enforcement of rules and procedures from the perspective of key informants [23] (pp. 86,87). The goal was to examine legislative barriers to and enablers of the implementation of TB-related laws in healthcare facilities. Our specific aim was to answer two questions: (1) What are the existing laws, regulations, and policy frameworks that provide for rights and obligations related to occupational TB in health workers in Mozambique? (2) What do key informants perceive as legislative barriers impeding the implementation of TB-related laws? 

### 1.1. Country Context

Mozambique is a low-income country located in Sub-Saharan Africa, estimated to have 31 million people as of May 2020 [26]. Mozambique achieved independence in 1975 after decades of armed conflict against Portuguese colonial rule, followed by 16 years of civil war. It was only in 1992, with the signing of the General Peace Accord, that Mozambique started to experience a relatively peaceful transition of governments. 

Despite considerable development progress since the Peace Accord, Mozambique remains in the lowest category of the Human Development Index (HDI) as of 2019 [27] and lags behind its peers in important human and social development indicators (ranked 180 out 189) [28]. Mozambique, for instance, suffers from a large communicable disease burden with high death rates, severe shortage of health workers (4.5 workers/10000 people), and unequal distribution of resources among provinces [29]. 

The health system consists of four interrelated sectors: (1) the public sector—or the National Health Service; (2) private for-profit sector; (3) private non-profit sector; and (4) community-level sector (or traditional medicine) [30]. The public healthcare sector, the focus of our study, is the main provider of medical services nationwide [12]. This sector is organized into four levels of care: level I: community health centres (primary health care and entry point); level II: general hospitals (clinical conditions, complications, and emergencies); level III: district level hospitals (specialized curative care); and level IV: central level and specialized hospitals [31]. TB treatment and control are integrated into all levels of care.

Health resource allocation to provincial and district governments is the responsibility of the Health Ministry [32]. The Ministry’s budget is provided by the central government as a percentage of the country’s annual budget and complemented by foreign assistance through collaborations forged directly between the Health Ministry and international organizations and/or donor nations (e.g., Global Fund). Healthcare funding varies annually and is subject to considerable controversy with respect to resource prioritization and allocation [31,33].

### 1.2. Research in Context

IPC offers a hierarchical approach of administrative, environmental, and respiratory protection controls [16]. Previous research has provided generally accepted evidence of the effectiveness of the package of IPC measures in preventing exposure and reducing the risk of TB infection in healthcare settings [34]. Studies have also shown that inadequate implementation is a problem in low- and middle-income countries LMICs [18,19]. This includes Mozambique, where, inter alia, statutory gaps, such as lack of legally established employer responsibilities, have been identified as a barrier [10]. Other than Brower et al. [10], there has been little systematic research into the legislative basis in Mozambique or other countries regarding the protection of health workers from occupational TB, and scant literature available for comparative research [24,35]. 

In this paper, we draw upon public health law and human rights literature to consider how enabling legal environments are needed to reduce the burden of disease and to provide adequate delivery of care and support to affected people [21,36,37]. Laws and regulations provide frameworks to guide action and to hold governments to account for any act or omission [38,39]. This literature, furthermore, has provided insights into the nature of vulnerability to TB infection and the burden of the disease (e.g., among the poor and people living with HIV/AIDS), as well as evidence that actions that integrate human rights contribute to effectively reducing TB infection [40,41,42,43,44].

In 2018, the Political Declaration on the Fight Against Tuberculosis following the United National General Assembly high-level meeting on tuberculosis reaffirmed the vulnerability of health workers as a group at increased risk of TB [40,45]. The Declaration [45] calls for immediate and effective actions to protect, promote, and realize the rights of health workers. Further, the right to health alongside international labour standards entitle health workers to protection against occupational diseases, including occupational TB [17] (s. 1.3.5). The ILO’s Resolution no. 194 [17] and international agreements, such as *the International Covenant on Economic, Social, and Cultural Rights* [46] (s. 6.2) together with the International Labour Organization’s *Convention on Health and Occupational Safety C155*, establish obligations on employers to implement the means for safe workplaces—at the very least to ensure that workers have proper protective gear [47]). 

Despite these specifications, references to the health workforce in the human rights literature primarily address efforts to improve care for TB patients, generally identifying health workers’ responsibility either as instruments (to provide adequate service) [48] or as advocates (to promote and support patient’s rights to adequate care) [38,44]. For example, human rights-based responses in Mozambique have focused largely on training health workers on issues of discrimination, confidentiality, and medical ethics to improve service provision to patients [8] (pp. 53, 57,58).

In response to the call from international agencies for integrated efforts to address occupational TB in low-income, high TB burden healthcare settings [45], our aim in this paper was to examine barriers to implementing IPC and occupational health and safety (OHS) through a lens of law and human rights. In line with this integrated approach, we include the legal codification of responsibilities respecting the duty of care, as well as an adequate rights and obligations framework to guide implementation of interventions.

## 2. Materials and Methods 

### 2.1. Study Design 

The present study was developed within an established collaboration linking three sets of institutions: Mozambican government health units, international agencies, and research institutions in Mozambique, Canada, and South Africa [49]. The initial project focused on evaluating a tool, termed HealthWISE, which was being implemented to assist healthcare workplaces in Mozambique, South Africa, and Zimbabwe to identify and address common occupational health hazards using existing resources. Team members in Mozambique identified the absence of a national policy tailored to protect health workers as a major barrier to effective implementation of protective practices. 

Mozambique’s current national TB policy [50] provides guidelines for IPC in congregate spaces in general and includes a limited section on healthcare settings but lacks a focus on occupational health and health workers. With a view to assisting the development of a national policy that provides specific guidelines related to occupational TB prevention among health workers [51], we designed the present research to examine the country’s existing legal frameworks and barriers to implementation. In the tradition of “law and society” scholarship (i.e., the interrelationship between law and social context and impact) [52,53], we applied an interdisciplinary approach that included legal analysis and case study qualitative methodology, as detailed in the following two sections. 

### 2.2. Constitutional and Regulatory Framework Review and Analysis

The legal aspect of our study draws on analysis of regulatory frameworks to examine the rights and obligations in respect of occupational TB and workers’ rights in Mozambique’s public healthcare setting. Given Mozambique’s civil-law legal tradition (i.e., written law, such as codes, statutes, and regulations, is the main source of law as opposed to judicial decisions as in common law legal traditions), we focused primarily on binding frameworks: laws and regulations. Judicial decisions were not included, as these are not considered a source of law in Mozambique’s civil-law legal tradition. 

We analysed twelve relevant legal documents (Table 1) following Mozambique’s hierarchy of law in relation to TB and occupational health in health workers, namely the Constitution, laws, regulations, international and regional treaties, and national policies. 

Legal interpretation methods (i.e., dictionary meaning, grammatical structure, and statutory context) and content analysis of international treaties were employed for analysis [54] (pp. 46–48). The legal interpretation, done by a Portuguese-speaking legally-trained member of our research team (R.G.), began by examining how the Constitution establishes rights and obligations relevant to the prevention and management of TB in health workers. Next, the analysis focused on the regulatory framework, policies, and international commitments employing a similar legal interpretation approach and content analysis, with a focus on how rights and obligations have—or have not—been realized.

### 2.3. Workshop and Policy Discussion Group Observations 

Following the law and society approach—where the implementation of laws and rights must be understood in social context—we observed a one-day-workshop in Maputo, Mozambique, on 16 November 2018, and a separate policy discussion group convened on 20 November 2018. The workshop gathered 30 participants, including health workers and managers (members of the local team), international organization representatives, and researchers in TB and law (members of the international team), as well as health and labour policy makers and government officials (involved in policy development). The workshop, facilitated by local team leaders and the International Labour Organization (ILO)’s country representative, was convened with two related goals in mind: The international team presented current knowledge on IPC and OHS approaches internationally, and the local team presented existing occupational operational procedures and practices at the three facilities that were participating in the HealthWISE project [49].

As part of the research design, we organized follow-up small discussion groups guided by questions to capture participants’ experience of facilitators of and barriers to legal implementation. Workshop participants were invited to participate in the small discussion groups; 15 individuals (out of 30) participated in the discussion groups. Participants were divided in two groups: one focusing on challenges for implementation of protective measures at the local level, and the other group focusing on legal implementation challenges more broadly. Groups were assigned according to participants’ roles and responsibilities at work. This paper reports the findings of the policy discussion group; findings on implementation challenges of protective measures are reported elsewhere [49]. The policy discussion group gathered five participants, including two health managers, one Labour Ministry staff member, one health worker, and one representative of the ILO (who also facilitated the discussion). The group discussed existing laws and policies, the focus of our paper.

Portuguese was the official language of the workshop and policy discussion groups. The first author (R.G.) carried out observations and took detailed notes. Both the workshop and policy group were audio recorded, and a content analysis approach was used to analyse the data, with R.G. thematically coding the observation data from the transcriptions. There was iterative discussion and agreement among team members of emergent themes and findings regarding facilitators of and barriers to legal implementation of the country’s laws, as well as what was needed to develop a policy likely to be successfully implemented in Mozambique. The workshop and policy discussion group data were combined for this purpose to maintain the anonymity of participants in the small policy discussion group where the source of comments could be identifiable. 

## 3. Results

### 3.1. Worker Rights, State Responsibilities, Workers’ Compensation in Mozambique

We analysed specific provisions of legal documents establishing rights and obligations relevant to occupational TB and health workers. Table 2 provides excerpts of provisions that are binding upon the government (i.e., employer). 

At the apex of the legal system is the Mozambique Constitution adopted in 2004 [55]. It formally establishes worker rights, freedoms, and guarantees (Table 2, row 1). This includes the right to workplace health and safety and the freedom to form trade unions. As a rule, any law which contradicts constitutional provisions can be considered unconstitutional and regarded as invalid. However, the exercise of worker rights and freedoms depends on enabling legislation that establishes responsibilities and steps for implementation. 

The Mozambican Congress passed two pieces of enabling legislation giving effect to rights and freedoms of workers. Specifically, in 2014, Congress passed enabling legislation that determined the steps for unionization in the public sector, namely the Trade Unions Act [56], which reaffirms workers’ constitutional rights to form and to join trade unions, as well as to collective bargaining for the protection of their work-related interests. Relevant to occupational TB, the *Trade Unions Act* provides for worker participation in the elaboration of working conditions and workplace safety (Table 2, row 2). The legislation provides for collective bargaining at facility and all government levels. However, the legislation provides no specific elements and mechanisms for public servants’ participation in any existing governmental Occupational Safety and Health Committee.

In Mozambique, public sector workers fall under the Ministry of Public Administration and Public Service and under the *Public Servants and Public Agents Act 10/2017*, which regulates relations in the public sector [57]. (Employment relations within the private sector fall under the *Employment Act 23/2007* [58] and industry specific laws; employment relations in the private sector are beyond the scope of our study). The *Public Servants Act* [57] reaffirms workers’ constitutional rights to unionization as above, and to workplace health and safety to be exercised under specific legislation (Table 2, row 3). The Act charges the federal government with the promotion of continuing education and engagement of workers in “collective methods of work management” and requires the government to develop specific frameworks that define further responsibilities and steps for implementation. 

With regard to regulation, the *National List of Occupational Diseases* for compensation is relevant [59]. TB is not included in the national list, and TB in health workers exposed to patients with TB is therefore not presumptively considered a compensable occupational disease in Mozambique. Under the country’s legal regime, compensation may be payable for work-related diseases not listed in the occupational diseases schedule provided that the worker proves the link between the disease and the workplace (Table 2, row 4) [60]. As such, this shifts the onus of proof onto the worker and involves a burdensome process. 

It is worth noting that while the private sector is subject to the same compensation regime as the public sector (Table 2, row 4), the legislation regulating employment in the private sector is more comprehensive, establishing, inter alia, thorough responsibility and accountability frameworks [58]. In addition, the private sector legislation provides that the statutory interpretation of its provisions should be in accordance with international human rights principles and best practices in the field [59]. In theory, this would allow the recognition of TB as an occupational disease in healthcare settings in the private sector. This interpretation standard is not part of the *Public Servants Act* [57]. 

### 3.2. International Treaties

According to the Mozambican Constitution [55], international and regional treaties come into full force once ratified by Congress, whereupon the government must ensure that all laws, policies, and programs in the country conform with international agreement, including adopting required legislation. Mozambique has signed, ratified, or acceded to several international and regional treaties relevant to the protection of workers, with emphasis on the Universal Declaration of Human Rights [61] (Arts. 23.1, 23.4), the International Covenant on Civil and Political Rights [62] (s. 22.1), and the African Charter on Human and Peoples’ Rights [63] (ss. 10, 15). These treaties require the Mozambican government to adopt comprehensive legislation and practice that enable, inter alia, the enjoyment of safe working conditions and freedom of association. 

Furthermore, Mozambique has been a member State of the International Labour Organization (ILO) since 1976 [64]. The country has ratified a number of conventions [65], including all eight fundamental conventions and three (out of four) governance priority conventions [66]. Two conventions are particularly relevant to protect health workers from occupational TB, namely *C19—Equality of Treatment* (*Accident Compensation*) *Convention*, *1925* (*No*. *19*) [67], and *C144*—*Tripartite Consultation* (*International Labour Standards*) *Convention*, *1976* (*No*. *144*) [68].

Ratification includes an undertaking to adopt and ensure that new and revised legislation complies with and enables tripartite consultation for implementing international labour standards within the country (i.e., C144). It also includes an undertaking to report on the application of the conventions regularly. The last country report on C144 dates back to September 2018 and reported overall developments on regular tripartite dialogue throughout the country without, however, providing specific content and outcomes [69]. 

Another barrier to holding the government accountable for deficiencies in occupational safety and health among health workers is that Mozambique is yet to ratify the *C155—Occupational Safety and Health Convention, 1981 (N. 155)* [47] providing an international standard for safety and health at the workplace. Under the ILO’s Constitution, Mozambique, as a member State, is still required to report on measures undertaken toward implementation of any provision of a non-ratified Convention [70] (s. 19.7.b.iv). The same applies to steps undertaken to include TB in the Mozambican national schedule of occupational diseases as per ILO’s *Resolution No. 194—List of Occupational Diseases Recommendation* that classifies TB as an occupational disease [17] (s. 1.3.5).

### 3.3. TB Control Policies 

In 1979, the Mozambican government created the National TB Control Program with centralized management at the federal level and decentralized services at provincial and district levels. At the federal level, the Health Ministry is responsible for setting national priorities, developing regulations and policies, resource allocation, and partnership coordination. At the provincial level, provincial health authorities are responsible for coordinating and overseeing implementation, resource distribution, and providing technical and logistical support to district governments. At the district level, local authorities are responsible for service delivery (The two hospitals and healthcare center analyzed in this study are service providers under district administration, but also report to the federal government).

In 2013, the Health Ministry launched the *National TB Program Plan* to provide strategic and operational directions to all health authority levels in respect of the essential components of TB prevention and control [33]. Following international standards and guidelines [16,71], the Plan [33] establishes the respect for human rights as guiding principles for implementation (p. 36), although without elaborating on what those efforts entail in relation to IPC at healthcare facilities. Ambitious targets for healthcare facilities were established: a 75% implementation by 2016, and 100% implementation by 2018 [33] (p. 87). However, limited resources were allocated in the TB Plan to achieve such targets, namely 2.2% of the total health budget [33] (p. 92). 

The Health Ministry also published the *National Infection Control Policy for Congregate Spaces* that sets out the overarching policy context for the national TB response in healthcare settings [50], addressing its responsibility for surveillance and data management; program monitoring and evaluation; community-based awareness; stakeholder engagement and cooperation; budget planning and allocation; and provision of technical assistance, training, and capacity building. In recognition of the increased risk of TB infection at the healthcare workplace, the policy sets out actions to protect health workers in healthcare settings, including supporting cross-governmental collaboration, engaging in targeted research, and conducting TB surveillance in health workers. Healthcare facilities are responsible for IPC implementation, including management, administrative, environmental, and personal controls, with administrative measures detailed in a written TB infection and prevention control plan to achieve accelerated laboratory diagnosis and TB treatment for health workers. The policy, however, provides a generic framework for IPC controls in healthcare settings for the protection of workers, patients, and the public, without defining specific employer responsibilities to create and maintain a safe and health workplace for health workers.

### 3.4. Key Informants’ Perceptions of Worker Rights and Barriers to Implementation 

Despite the existence of the national policy described above, we identified major legal gaps as perceived by workshop and policy discussion group participants as barriers to effective realization of health workers’ rights to be protected from TB at their workplace. Four inter-related themes emerged: legal classification of occupational TB; self-disclosure, privacy work-related stigma and discrimination; unionization; and limited state resources. First, all participants agreed that not having TB officially recognized as an occupational disease was a substantial barrier. The general view was that government acted in such cases only when compelled by law, with one manager explaining, “codification is the only way that governments get things done in this country.” There was therefore “the need to amend the National List of Occupational Diseases” (issued in 1957), which does not include occupational TB. Under Mozambique’s compensation regimen, the addition of TB to the list would entail the presumption that the disease in health workers exposed to patients with TB was occupational and hence would create a legal obligation upon the state to compensate workers who were infected on the job. Such presumptive compensation would also exert pressure upon governments to implement and maintain the health and safety of the workplace “in order to not have to pay compensation.” 

The second emergent theme was reluctance among health workers to disclose a TB diagnosis owing to fear of stigma and workplace discrimination. One participant explained that Mozambique has a law requiring TB case-reporting to health authorities, but there was no law mandating TB case-reporting to employers. In fact, one manager noted that in her experience, workers typically do not disclose TB diagnosis to management, although noting, “if a worker misses more than a few days of work it is likely that everyone will guess what is happening.” The general sense was that the lack of privacy enabled pervasive "gossip" at the workplace. One health worker explained: “people will even think that the person on sick leave [when TB is guessed, but not disclosed] is also HIV positive, because of the connection between TB and HIV.” Another participant concluded, “... there are no specific rules for disclosure or for workers’ privacy, only a lot of gossip ... something needs to be done about the gossip.” 

Particularly problematic from the perspective of managers was that if a worker who is sick with TB does not disclose the diagnosis, the "health worker zero" transmission goes unidentified, and the infection is not promptly addressed. This is especially challenging, as a manager explained, “... workers usually seek TB treatment elsewhere. Employers are not notified that the worker is being treated for TB. We guess, but it could be treatment for any other disease, really. TB then goes unaddressed in our unit.” Whether, and especially how, employment disclosure should be achieved raised a heated debate among participants. On the one hand, managers were of the opinion that special legal attention should be given to a mechanism for mandatory disclosure and testing. On the other hand, health workers were reluctant to accept mandatory disclosure of a TB diagnosis. Workers were emphatic about the need for legal attention to where treatment could be sought, reporting a strong preference for healthcare facilities other than their own workplace. Managers, however, vehemently disagreed with an option of sick workers seeking treatment outside their own workplace, with one manager stating, “... if we allow that [non-disclosure and treatment in other hospitals], we will never combat occupational TB and address TB-related stigma in our hospital. We all lose.” 

The prevailing opinion was that while greater professional confidentiality was needed, this should be supported by regulation setting out the rules of privacy, notification requirements, and responsibilities for the implementation of education and awareness campaigns. 

The third theme was the lack of trade unions for public servants associated with “an opportunity to be heard”. Participants generally agreed that trade unions created important spaces for action. A few participants complained about the lack of opportunities to pressure government to improve health and safety at work, and to lobby for insurance coverage for occupational health. There was, however, an incorrect perception that there was no enabling legislation providing for the formation of trade unions. One participant reported the existence of a nurses’ association, explaining that the role of the association was mainly to assist its members with grievance procedures rather than exerting pressure over decision-making. Participants were also unaware of any mechanism to monitor law, policy, or regulation reform in the country and of any training to strengthen knowledge and awareness about laws and policies, including in relation to workplace health and safety other than a few pamphlets hanging on walls. 

With respect to “an opportunity to be heard”, informants showed some interest in the status of Mozambique’s Treaty ratification after the presentation of the International Labour Organization (ILO)’s representative (e.g., *C144—Tripartite Consultation (International Labour Standards) Convention, 1976* [68], and *Convention C155—Occupational Safety and Health Convention, 1981* [47]). However, there was no engagement with what such conventions would mean in practice in relation to tripartite labour management toward influencing better working conditions. The discussion about unionization also brought to the fore considerations about the regulatory disparity between the public and private healthcare sectors. For example, one participant in the discussion group showed a binder with a compilation of laws regulating occupational health in the private sector, highlighting the regulatory gaps in the public sector, “…we [public servants] are very disadvantaged with respect to occupational health and organized labour in relation to the private workers. There is little to nothing about this for us.” One participant noted, however, that Mozambique’s private healthcare sector is small and incipient, with insufficient legislation and poor unionization. 

The fourth theme was one of limited resources in the public sector, with one health worker noting, “we cannot compare the public and the private sector ... the private has money, the public has not.” This discussion turned into debates about implementation challenges owing to resource constraints, with one government official elaborating, “…the public budget is in deficit ... that is why we have problems in Mozambique ... donors cut external resources and the state faces challenges to implement national health plans. So, there is no resource to do many things.” However, different views were expressed, e.g., “‘we don’t have money’ is the classic answer when Mozambique’s governments want to justify omission.” Another manager reiterated that implementation challenges were due to a lack of legislation rather than resource constraints, and that “… what we need is for occupational TB to become law… then the State [employer] has to come up with resources and ways to address stigma.”

## 4. Discussion 

Several contributions emerged regarding legislative barriers and enablers of the implementation of TB-related laws and policies to protect health workers in low resource, high TB burden settings.

Despite being a low-income country, Mozambique has a fairly well-developed structure of laws and policies for protection against occupational TB in healthcare settings, which, as recognized in the literature [10,24,25,38], is central to improved implementation of measures to protect health workers from TB. In particular, prescriptive regulations that clearly establish responsibility frameworks were seen as necessary to improve implementation; and the lack of codification of occupational TB was viewed as a barrier to the effectiveness of prevention measures, even more so than resource shortages such as supplies, equipment, and infrastructure. 

These perceptions were explicitly associated with the classical conception of law as command backed by threat [72], rather than “softer” conceptions of law as providing a framework for action, as expressed in the human rights literature [38]. The latter approach has shown positive results in the global arena in regard to wider access to medicines and increased funding for TB domestically [73] (pp. 215–244). However, while our legal analysis identified applicable legislation and policy, Mozambique’s civil-law legal system (i.e., based on codes and regulations that use providing detailed and mandatory language) may have led to the belief, especially among civil servant respondents, that nothing significant will change at the workplace until responsibilities for TB in health workers are assigned, codified in the law, and enforced. 

In line with several studies, this study suggested that a lack of attention to TB-related stigma is a barrier to protection of health worker against TB [38,74,75]. Work-related stigma in Mozambique is associated with reluctance of workers to disclose their TB diagnosis, and anxiety about privacy and confidential medical treatment. This finding extends the analysis of Brouwer et al. [10] of TB protective practices in Mozambique, in which the authors identified the need for “management responsibility” in respect of personal controls, equipment provision, and motivation of health workers to fully adhere to protection measures. Our findings identified the need to establish clear responsibilities and action plans for training programs and campaigns addressing stigma at the workplace. 

A 2013 study conducted in three Mozambican provinces [76] found that TB-related stigma and discrimination were prevalent in healthcare settings and at the community level more broadly. In response, a program focusing on health workers’ training in human rights and ethics components for improving the care of TB patients was developed [8] (pp. 49,50). As reported by the Global Fund [8], the program was cut short owing to deficient funding to conduct the planned training. However, the human rights and ethics of care content in the training was directed exclusively at the care of TB patients with health workers framed either as “instruments” or “advocates” to improve the quality of care [8] (pp. 49,50). This approach is reflective of the human rights literature to date [44,48]. 

Finally, this study explored health workers’ knowledge and understanding of existing laws, regulations, and polices related to TB and occupational health. Legal literacy, understood here as the knowledge of the content of rights and law, as well as the processes through which laws and policies are developed, is a basic requirement of efforts to promote, protect, and realize human rights worldwide [40,44]. Our results suggested low levels of legal literacy, especially with respect to the content of workers’ rights and mechanisms for monitoring and reforming laws and regulations. To illustrate this point, the lack of enabling legislation providing for trade unions in the public sector was reported by a number of participants as a legislative barrier to collective bargaining for improved employment conditions. Our legal analysis, however, determined that Mozambique’s Congress had in fact passed enabling legislation in 2014 [56]. The legislation, furthermore, also established mechanisms for collective methods of work management [57]. However, no organized labour structures were in place four years later. 

Poor dissemination of the content of laws, an environment in which the employer is also the regulator, and entrenched belief that limited resources should be directed to service delivery rather than workplace safety, may be among the reasons for the lack of efforts to achieve collective action. A discussion of unions is beyond the scope of this paper; our example here is intended to underline the fact that rights awareness among health workers is lacking. It is increasingly accepted in the human rights literature and by international agencies that integration of human rights considerations into TB prevention and control efforts is needed for the implementation of TB-related laws and policies to protect health workers [40,45,71]. A human rights approach, inter alia, provides a framework for enabling health workers to participate in directing the policies, programs, and practices that affect their vulnerability to occupational TB. 

We have previously argued that the worker-centred lens is needed for improved protection of health workers against occupational TB [77] and have called for greater integration of IPC and occupational health and safety (OHS) [77,78,79]. Besides the core components of IPC, the OHS approach includes an emphasis on worker screening, treatment, and disability management; confidential occupational health services; and education about occupational risks and rights [78]. It also assumes a legal and political framework for consultation and negotiation with those affected [77]. 

Our study has limitations that should be noted. Although it was aimed at understanding how law and regulatory regimes can improve occupational health and safety of workers [80,81], the stakeholder engagement component was limited and did not include health worker associations. It is also possible that the mix of stakeholders who took part in the meetings resulted in a narrower range of expressed opinion than would have been the case had these groups met separately. 

## 5. Conclusions

This is the one of the first studies we are aware of examining how deficiencies in legal and governance frameworks might hinder implementation of TB-related laws and policies to protect health workers in low resource, high TB burden settings. In the Mozambican context, these deficiencies include a lack of classification of TB as an occupational disease in health workers, and failure to provide regulation for occupational health and safety, which would clearly define employer responsibilities, including dealing with issues of privacy and stigma. Lack of health worker understanding of their rights under international treaty and local law, and of their collective rights to influence law and regulation, adds to their vulnerability.

Further empirical legal research is needed to provide evidence on the effects of TB-related laws, regulations, and policies in reducing occupational TB in health workers in low resource settings. Research is needed on ways to support country stakeholders, including via the ILO, in putting in place needed occupational legal and governance frameworks. Efforts to protect health workers from TB would benefit from an analytical frame within which to engage with the realisation of workers’ human rights to a safe and stigma-free workplace as a health system goal in its own right. More generally, understanding among stakeholders of the inter-relationships between different rights is more likely to lead to calls for political action, including for needed legislative and/or regulatory change. 

## Figures and Tables

**Table 1:** List of analysed legal frameworks.

Frameworks	Content Analysed
*Federal Constitution, 2004*	Workers’ rights and freedoms
*Trade Union Act No. 18, 2014*	Unionization of public servants
*Public Servants and Public Agents Act No.10, 2017*	Labour relations in the public sector
*Accidents at Work and Occupational Diseases Regulation No. 62, 2013*	Workers’ compensation scheme
*National List of Occupational Diseases, 1957*	Standard for compensable diseases in Mozambique
*Universal Declaration of Human Rights*	Workers’ rights
*International Covenant on Civil and Political Rights*	Workers’ freedoms
*African Charter on Human Rights and People’s Rights*	Workers’ rights and freedoms
*Constitution of the International Labour Organization*	Report
*Convention Tripartite Consultation (International Labour Standards) Convention No. 144, 1976*	Tripartite negotiations
*TB Strategic Plan 2014–2019, extended to 2023*	TB control plan
*Control of TB in congregate spaces policy and national plan for infection control for tuberculosis in sanitary units and congregate environments of Mozambique*	TB control plan for healthcare settings

**Table 2:** Statutory provisions related to occupational TB in health workers.

Frameworks	Provisions Analysed
Constitution of the Republic of Mozambique [55]	Art. 84 (1) Everyone has the right and duty to work. Art. 85 (2) Every worker has the right to protection, health, and safety at the workplace. Art. 86 (1) All workers shall have the freedom to organize professional associations or trade unions. Art. 86 (4) The law shall regulate the formation, merger, alliance, and dissolution of trade unions and professional associations, and the law shall lay down the guarantees to their independence and autonomy in relation to employers, the State, political parties and churches, and religious denominations.
Trade Union within Public Sector Act [56]	Art. 7 (1) Public servants have the following rights under this law: (a) participate in the formation of trade union; (b) join trade union; (c) relinquish trade union membership. Art. 27.1 The public administration and trade union associations shall give priority to social dialogue for workers participating in the process of defining working conditions, elaboration of public policy, and defence of social and work-related interests. Art. 27.2 The dialogue referred in the previous section occurs through collective bargaining and consultation according to this legislation. Art. 28. Collective bargaining takes place every five years, … extraordinary negotiations may take place whenever needed, and consultations may take place on a permanent basis. Art. 28.1 Collective negotiations related to wages takes places on an annual basis. Art. 29 The subjects covered by collective negotiations are: (g) working conditions, the prevention of work hazards, and workplace safety. Art. 29.1 Subjects covered by consultation [carried out by governments] include: (f) ratification processes of international labour conventions from the ILO and other international agencies. Art. 31 Negotiating parties include trade unions’ representatives, and the respective level of government or sector.
Public Servants and Public Agents Act [57]	Art. 47 Workers shall be guaranteed: (1) (c) adequate conditions of hygiene and safety at work, as laid down by specific law. (1) (y) freedom to form and operate trade unions, as laid down by specific law. Art. 45 (2) The state shall be charged with promoting: (e) continuing education for development of workers. (g) collective work management practices, practicing dialogue with workers for improving working conditions, and fostering workers’ integration into institutional development processes.
Accidents at Work and Occupational Diseases Regulation	Art. 20 (2) Occupational diseases are considered in the National List of Occupational Diseases. (TB is not included in the list) Art. 20 (3) If a disease is not included in the National List of Professional Diseases, but there is medical evidence of causal relationship between the disease and the working conditions, the worker is guaranteed the right to reparation under the terms of this Regulation.
